# Risk and Protective Factors for Problematic Drinking in Early Adolescence: A Systematic Approach

**DOI:** 10.1007/s10578-019-00925-1

**Published:** 2019-08-29

**Authors:** Johan Isaksson, Mary Schwab-Stone, Andrew Stickley, Vladislav Ruchkin

**Affiliations:** 1 Child and Adolescent Psychiatry Unit, Department of Neuroscience, Uppsala University, 751 85 Uppsala, Sweden; 2 Child Study Center, Yale University Medical School, New Haven, CT 06520 USA; 3 The Stockholm Center for Health and Social Change (SCOHOST), Södertörn University, Huddinge, Sweden; 4 Department of Preventive Intervention for Psychiatric Disorders, National Institute of Mental Health, National Center of Neurology and Psychiatry, Tokyo, Japan; 5 Säter Forensic Psychiatric Clinic, Säter, Sweden

**Keywords:** Alcohol problems, Early adolescence, Depression, Conduct problems, Ethnicity

## Abstract

Alcohol use during early adolescence is associated with other risk behaviors as well as future health problems. Within the design of a larger prospective research program, a cohort of U.S. inner-city sixth-grade students (N = 1573, mean age = 12.10) were assessed and reassessed in the seventh-grade. Self-reported information was obtained on problems related to alcohol, fixed markers of risk (e.g. sex, age, SES), individual and interpersonal factors (e.g. internalizing and externalizing symptoms) and contextual factors (e.g. substance availability). Alcohol-related problems in seventh grade were foremost predicted by individual and interpersonal factors in the sixth grade including depressive symptoms, conduct problems, a decreased perception of wrongdoing, and affiliation with delinquent peers. In addition, alcohol use in the sixth grade and being of Hispanic or White ethnicity was also associated with subsequent alcohol-related problems. Interventions should be directed towards assessing and treating individual risk factors such as depression and externalizing symptoms.

## Introduction

Alcohol use is common during adolescence with most teens having consumed alcohol before 15-years of age [1]. While for many adolescents, the use of alcohol is limited to normal experimentation, a substantial proportion of adolescents experience alcohol-related problems, and alcohol is regarded as the main risk factor for disease in young people, contributing to 7% of cause-specific disability-adjusted life-years [2]. Approximately 22% of adolescents report binge drinking [3] and up to 15% abuse alcohol, resulting in poor school performance, interpersonal and legal problems [4]. Furthermore, problematic drinking during adolescence often continues into adulthood and predicts future alcohol dependence [5], with drinking during early adolescence, i.e. 10–14 years-of-age, being associated with an especially increased risk for alcohol abuse and dependence [6, 7], as well as a higher prevalence of risky sexual behavior and early cigarette and marijuana use [8]. Alcohol abuse may also have a damaging effect on the adolescent brain, which undergoes significant neural development during early adolescence [9].

Given the potentially damaging effects of alcohol misuse during early adolescence, there is an imperative to investigate risk and protective factors associated with problematic drinking from a perspective of prediction and prevention. Most studies on this subject have been cross-sectional, although a growing number now use a prospective design in order to disentangle risk and protective factors while also applying different theoretical frameworks [10]. These different frameworks often cover a broad range of variables, which can be grouped into fixed, individual, interpersonal, and contextual risk and protective factors [10, 11]. To date, however, this systematic approach has mainly focused on alcohol use and age of debut, rather than the detrimental consequences of alcohol use, and the research has mainly been undertaken among late adolescents and young adults, or included a large age-range [11, 12]. Review studies have highlighted a vast number of risk/protective factors associated with alcohol use and misuse, including fixed factors, such as being male and/or of White ethnicity; intrapersonal risk factors, including symptoms of depression and a variety of externalizing problems, such as conduct problems, sensation seeking behavior and hyperactivity, as well as a low perception of risk; interpersonal risk factors such as alcohol-using peers and problematic family relations where family conflicts have been proposed as a risk factor whereas parental warmth and support have been shown to decrease the risk for substance use; lastly, contextual risk/protective factors include perceived social norms and increased availability of alcohol [11, 12].

During early adolescence, most longitudinal studies have emphasized the role of externalizing problems for early alcohol consumption [13, 14], alcohol-related problems such as heavy drinking and being drunk [14], and for alcohol abuse and dependence [15]. Studies on internalizing symptoms have produced somewhat mixed results, with the report of an association between depression at age 11 and alcohol problems at age 14 [14], whereas other research has found no association between internalizing psychopathology and alcohol use disorder onset [15], or even suggested a protective effect against early substance use [13]. Since the use and misuse of alcohol becomes more normative with increasing age, it has been suggested that externalizing symptoms such as impulsivity and antisocial behavior may have a larger impact at early ages, whereas internalizing symptoms such as anxiety problems may be protective during these early years [16]. Thus, the reported findings on depression as a unique predictor for substance use among late adolescents and young adults [17], may not necessarily be valid in early adolescence.

Previous studies have also suggested that in order to elucidate the impact of externalizing and internalizing problems on alcohol-related problems during early adolescence, there is a need to also adjust for a large variety of risk and protective factors [10, 11]. For instance, the child’s sex may moderate the association between psychiatric symptoms and alcohol use, with e.g. reports suggesting that an association between depressive symptoms and problematic alcohol use is evident for girls but not boys [18, 19]. The influence of ethnic origin on alcohol consumption also seems to vary by age, with reports of more alcohol use among Hispanic students in the eighth grade and no difference between White and Hispanic students by the tenth and twelfth grades [20]. Age differences in relation to problematic alcohol use may also be of relevance for contextual factors, and in line with this, a review reported mixed results regarding neighborhood effects on adolescent alcohol use, although factors such as the density (availability) of liquor outlets in communities seem to be of greater importance during early adolescence compared to late adolescence [21].

To summarize, while a vast number of risk and protective factors have been identified for both alcohol consumption and the negative consequences of alcohol use, there are a limited number of prospective studies on younger adolescents that address problems related to alcohol use, such as interpersonal (getting into fights with friends, etc.) and social problems (neglecting responsibilities, problems with schoolwork, etc.). Moreover, there is limited research utilizing a more systematic approach that includes a broad range of factors in the same model. Within the design of a prospective research program, following a large cohort of U.S. inner-city sixth-grade students, we saw an opportunity to include a multitude of variables in the same model in order to identify factors that may be important for problematic drinking and consequently may be targeted in early prevention efforts. The sample represents a highly vulnerable group of early, predominantly ethnic minority adolescents, with a low socioeconomic background. We hypothesize that factors such as externalizing problems, but not internalizing problems, a low perception of risk, alcohol-using peers, a lack of parental warmth and support, and increased alcohol availability will predict problematic drinking. In addition, we intend to explore whether any of the risk/protective factors for alcohol-related problems differ by ethnicity or sex.

## Methods

### Participants and Procedure

Data came from two waves of a large-scale longitudinal study of self-rated risk and protective factors for problem behaviors, the Social and Health Assessment (SAHA) [22, 23]. The survey was administered in the public-school system in the Northeastern U.S. Parents were informed of the survey prior to its administration, that participation was voluntary and thus offered the opportunity to decline participation. In addition, students were read a detailed assent form outlining their participation, assuring confidentiality, and then asked for their signature to indicate assent. The refusal rate for parents and children was less than 1%. In total, 1948 reports were collected. After excluding students who lacked data for the outcome variable (n = 375), the study sample comprised 1573 students between the ages of 11–13 (Mean age = 12.10, SD = 0.73; 52.7% girls) who completed the survey during the spring of year 1 and were followed-up in the spring of year 2 (Mean age = 13.10, SD = 0.74). The participants were predominantly non-Caucasian: 59.3% African-American, 27.7% Hispanic, 11.1% Caucasian, and 1.9% other, an accurate reflection of the local public-school population. Those excluded did not differ from the study participants in prevalence of problems related to alcohol.

The survey was administered to students in groups in their classes by trained personnel affiliated with the school district and/or university undertaking the study. One administrator read the survey aloud to students while the students followed along. In addition, a second administrator was available to answer any questions from the students. The entire administration procedure typically lasted 1 h. Surveys were administered in English or Spanish, as appropriate, and a makeup administration day was scheduled for each school within 1 month of the initial administration for students who were absent. A detailed description of the methodological aspects of the study is available in previous reports [22, 23]. The study was approved by the Yale School of Medicine Institutional Review Board and by the local Board of Education.

### Measures

#### Problems Related to Alcohol Use

Alcohol-related problems at seventh grade were measured with a ten-item scale that asked the respondent how often during the past year he/she had experienced problems as a result of alcohol consumption, e.g. being drunk, got into fights or arguments, had problems with schoolwork (Table 1). Items were answered using a four-point scale ranging from “0 times” to “6 or more times”. The Cronbach alpha for the total scale was 0.86. Scores were dichotomized (0 times, ≥ 1 time) so that a negative response for all items was scored as ‘0’, indicating no problems related to alcohol while a positive response on any item was scored as ‘1’, indicating alcohol-related problems.

**Table 1:** Prevalence of different types of alcohol-related problems in the past year by sex (N (%))

Drinking of alcohol caused any of the following	All	Boys	Girls	Chi square
Got into fights or arguments	167 (10.6)	85 (11.4)	82(9.9)	0.97
Got drunk	227 (14.4)	96 (12.9)	131 (15.8)	2.67
Had money problems	77 (4.9)	48 (6.5)	29 (3.5)	7.35**
Got into trouble at school	112 (7.1)	64 (8.6)	48 (5.8)	4.69*
Had problems with schoolwork	105 (6.7)	65 (8.7)	40 (4.8)	9.63**
Damaged friendships	93 (5.9)	45 (6.0)	48 (5.8)	0.05
Passed out	80 (5.1)	31 (4.2)	49 (5.9)	2.47
Couldn’t remember what happened	120 (7.6)	52 (7.0)	68 (8.2)	0.82
Got arrested	43 (2.7)	26 (3.5)	17 (2.1)	3.08
Rode in a car/vehicle driven by someone who had been drinking alcohol	174 (11.1)	79 (10.6)	95 (11.5)	0.28
At least one alcohol-related problem	447 (28.4)	208 (28.0)	239 (28.8)	0.15

*p < 0.05, **p < 0.01

#### Fixed Markers of Risk

Fixed markers included sex, age, ethnicity and living in a single parent family. In addition, eligibility for free or reduced lunch (if a student’s family income was less than 185% of the federal poverty guidelines) was used as an index of (low) socioeconomic status (SES) and was coded as 2 and 1, respectively.

#### Individual and Interpersonal Factors

These risk and protective markers were measured at sixth grade with a variety of different scales where higher scores indicated greater symptomatology (worse mental health/increased problematic behaviors) if not stated otherwise. In addition, having used alcohol at baseline was included as an individual risk factor, where the student rated lifetime consumption of three alcoholic beverages (beer, wine, and hard liquor) using a four-point scale, ranging from “never” to “more than a few times”. Based on the reports of alcohol use at baseline, two alcohol use groups were created: Abstainers (coded as 0) and Users (1).

Depressive symptoms were assessed with an adapted version of the Center for Epidemiologic Studies-Depression Scale [24] consisting of ten negative statements (e.g. “not liking oneself”, “loss of interest in other people or things”, “felt like crying”). The students reported on symptom presence in the past month on a three-point scale: “not true”, “somewhat true”, “certainly true”. The possible total score ranged from 0 to 20. Cronbach’s α for the scale was 0.85.

Anxiety symptoms were assessed with a 12-item scale [23] describing worrisome, preoccupying thoughts or unpleasant feelings about oneself or external stimuli (e.g. “I worry about other people liking me”, “I stay away from things that make me nervous”). The students reported on a three-point scale (“not true”; “somewhat true”; or “certainly true”) with the possible total score ranging between 12 and 36. Cronbach’s α for the scale was 0.84.

Posttraumatic stress was measured with the Child Self-Report Post-Traumatic Stress Reaction Index (CPTS-RI) consisting of 20 items corresponding to the diagnostic criteria of PTSD (e.g. “do you get scared or afraid because you think about bad things that have happened to you?”, “do thoughts or pictures of bad things that have happened to you come back to you, even when you don’t want them to?”), answered on a 5-point scale, from “never” to “most of the time”, with the total score ranging from 0 to 80 [25]. The Cronbach α for the scale was 0.88.

Conduct problems were assessed with six items from the Antisocial Behavior Scale [22]. The scale describes relatively mild behavior problems, such as staying out all night without permission or shoplifting. The students report on a five-point scale how many times they engaged in the problematic behavior, ranging from “0 times” to “5 or more times”, with the total sum score ranging from 0 to 24; The Cronbach α for the scale was 0.77.

Sensation seeking was measured using the Brief Sensation Seeking Scale [26], consisting of eight items (e.g. “I would like to explore strange places”, “I like to do frightening things”) where the students report on a five-point scale, from “strongly disagree” to “strongly agree”. The total score ranges between 8 and 40. The Cronbach α for the scale was 0.75.

Perception of risk was assessed with eight questions enquiring about the adolescent’s perception of the risk of harming themselves when engaging in potentially dangerous activities, such as substance use, gun carrying, dropping out of school, etc. [23]. Items were rated on a four-point scale, with the response alternatives ranging from “no risk” to “great risk”. The scale gives a total sum score that can range from 8 to 32 with a higher score reflecting a greater perception of risk. The Cronbach α for the scale was 0.95.

The perception of wrongdoing was assessed with eleven items [23] on “how wrong is it” to be involved in various antisocial activities (stealing, lying, damaging property, hurting someone badly in a fight, starting a fist-fight, etc.). Items were rated on a four-point scale with the response alternatives ranging from “not wrong” to “very wrong”. The total score could range between 11 and 44, with a higher score reflecting greater levels of disapproval of antisocial behavior. The Cronbach α for the scale was 0.88.

Affiliation with delinquent peers was evaluated with a nine-item scale [23], where scores ranged from 9 to 36, asking students about how many of their friends, ranging from “none” to “most or all”, were involved in risk taking behaviors, such as dropping out of school and smoking cigarettes. The Cronbach α for the scale was 0.87.

Parenting behavior was assessed with questions [23] enquiring about students’ perceptions of different aspects of parental practices. Parental warmth was measured with five items (e.g. “My parents or guardians… Are kind to me”, “Hug or kiss me”), parental involvement with six items (e.g. “asks about her/his life”, “gives good advice”), and parental control with eight items (e.g. “tell me what time to be home when I go out”, “want to know who I am meeting”). The students reported on a four-point scale, ranging from “never” to “often”. Cronbach’s alpha was 0.81 for the parental warmth scale, 0.75 for the involvement scale and 0.75 for the control scale.

School environment was assessed with the school environment and academic motivation scales [23], where school attachment was measured with five items (e.g., I like school; most mornings I look forward to going to school) and perceived teacher support was measured with eight items (e.g. “teachers are willing to help students”; “most of my teachers notice when I am doing a good job and let me know about it”). The students responded on a four-point scale on how true each of the statements is for them (ranging from “definitely not true” to “definitely true”), with the total score ranging from 5 to 20 for school attachment and 8 to 32 for perceived teacher support. Cronbach’s α was 0.77 for the school attachment scale and 0.67 for the perceived teacher support scale.

#### Contextual Factors

Contextual factors included five items from the Neighborhood Perception scale [23] enquiring whether participants had a negative perception of their neighborhood (e.g. “in my neighborhood there are problems because of racial differences”, “there is litter, broken glass or garbage on the streets, on sidewalks, or in yards in my neighborhood”). All items are scored on a four-point scale ranging from “definitely not true” to “definitely true”, with a possible total score range between 5 and 20. Cronbach’s alpha for the scale was 0.62.

Access to substances was measured with four items asking the respondent how easy it would be for him/her to get cigarettes, alcohol, marijuana or cocaine [23]. Responses ranged on a four-point scale from “very easy” to “very hard”, with the possible total score ranging from 4 to 16. Cronbach’s alpha was 0.81 for this measure.

### Statistical Analyses

All statistical analyses were performed using the Statistical Package for the Social Sciences (IBM SPSS version 25). Sex and ethnic differences in reports of alcohol-related problems were calculated with Chi square tests and a *t* test for the continuous scales. A Multivariate Analysis of Covariance (MANCOVA) was performed in order to assess the association between problems related to alcohol use in grade seven and the possible risk/protective factors in grade six, while also determining the sex- and age-specific contribution to any association. Thus, we used a 2 (reported, not reported alcohol-related problems) × 2 (sex) × 3 (age range, 11, 12 and 13 years) design for the ratings on risk/protective factors. We also adjusted for SES, single parent family, ethnicity and using alcohol at baseline. Those factors that were associated with alcohol-related problems were later included in a hierarchical multiple binary logistic regression model in four blocks; Block 1 adjusted for fixed factors; Block 2 additionally adjusted for use of alcohol at baseline; Block 3 also adjusted for individual and interpersonal factors; and in Block 4 contextual factors were also adjusted for. The results are presented as odds ratios (OR) with 95% confidence intervals (CI). Two-tailed tests with p-values < 0.05 were considered as being statistically significant.

## Results

In total, 447 (28.4%) of the children reported problems related to alcohol use in grade seven. As shown in Table 1, the most common reported problem was having been drunk (14.4%), followed by being in a car driven by someone who had been drinking alcohol (11.1%), and getting into a fight or argument with other people when drinking (10.6%). There were no sex differences in overall problems related to alcohol use, either for any particular problem or when examined as a continuous scale reflecting the magnitude of problematic use. However, at an item-level, more boys than girls reported that drinking during the past year had caused money problems, trouble at school, and trouble with school work. In addition, there were ethnic differences in alcohol-related problems (*χ*^2^= 14.59; *p *= 0.002), with 34.3% of Hispanic, 32.6% of White, but only 24.9% of African American students reporting any alcohol-related problems.

### MANCOVA

No sex- or age-specific effects were found for the association between problematic alcohol use and the risk/protective factors. However, there was a main effect for the overall problematic use of alcohol (Wilks’ lambda = 0.93; *F* = 6.76, *p* < 0.001, *η*^2^ = 0.07), where those with alcohol-related problems in year two had higher year one ratings for depression (*F* = 14.36, *p* < 0.001, *η*^2^ = 0.01), posttraumatic stress (*F* = 12.45, *p* < 0.001, *η*^2^ = 0.01), conduct problems (*F* = 52.42, *p* < 0.001, *η*^2^ = 0.04), sensation seeking (*F* = 16.21, *p* < 0.001, *η*^2^ = 0.01), and affiliation with delinquent peers (*F* = 44.13, *p* < 0.001, *η*^2^ = 0.03), as well as a lower perception of risk (*F* = 4.41, *p* = 0.036, *η*^2^ = 0.00) and wrongdoing (*F *= 57.88, *p *< 0.001, *η*^2^ = 0.04), lower ratings on parental control (*F *= 4.52, *p* = 0.034, *η*^2^ = 0.00), teacher support (*F* = 4.18, *p* = 0.041, *η*^2^ = 0.00), and school attachment (*F* = 10.10, *p *= 0.002, *η*^2^ = 0.01), and lastly, higher ratings on substance availability (*F* = 26.97, *p* < 0.001, *η*^2^= 0.02). In addition, for the risk/protective factors there was a main effect for having used alcohol before the sixth grade (Wilks’ lambda = 0.87; *F* = 14.23, *p* < 0.001, *η*^2^ = 0.14), sex (Wilks’ lambda = 0.95; *F* = 4.50, *p* < 0.001, *η*^2^ = 0.05), age (Wilks’ lambda = 0.95; *F* = 2.57, *p* < 0.001, *η*^2^= 0.03), White (Wilks’ lambda = 0.94; *F* = 6.29, *p* < 0.001, *η*^2^= 0.06) and Hispanic ethnicity (Wilks’ lambda = 0.96; *F* = 3.72, *p* < 0.001, *η*^2^ = 0.04), and SES (Wilks’ lambda = 0.96; *F* = 3.59, *p* < 0.001, *η*^2^ = 0.04).

### Adjusted Model (Logistic Regression)

In a hierarchical binary logistic regression analysis, that included only the risk/protective factors that differed between those reporting and those not reporting any problems related to alcohol in the MANCOVA, the final model explained 19.5% of the variance in problems associated with alcohol use, with individual factors explaining most of the variance, followed by use of alcohol at baseline assessment, fixed, interpersonal, and lastly contextual factors. As shown in Table 2, in the final model drinking-related problems in year 2 were predicted by having a Hispanic or White ethnicity, alcohol use at baseline assessment, symptoms of depression and conduct problems, a lower perception of wrongdoing and affiliation with delinquent peers.

**Table 2:** Results of a hierarchical binary logistic regression analysis predicting alcohol-related problems in early adolescents

	Step 1	Step 2	Step 3	Step 4 (final model)
	OR (95% CI)	OR (95% CI)	OR (95% CI)	OR (95% CI)
Sex (ref = females)	0.94 (0.75–1.17)	0.93 (0.74–1.16)	0.88 (0.69–1.13)	0.88 (0.69–1.13)
Age	1.46 (1.25–1.71)***	1.32 (1.12–1.54)**	1.19 (1.01–1.41)*	1.18 (0.99–1.40)
SES	1.05 (0.93–1.20)	1.06 (0.93–1.21)	1.04 (0.91–1.20)	1.05 (0.92–1.21)
White	1.48 (1.03–2.13)*	1.50 (1.03–2.17)*	1.54 (1.03–2.30)*	1.52 (1.02–2.27)*
Hispanic	1.55 (1.21–1.99)**	1.59 (1.23–2.05)***	1.60 (1.22–2.10)**	1.59 (1.21–2.08)**
Model R^2^	0.035			
Alcohol use year one		2.83 (2.47–3.57)***	1.83 (1.41–2.36)***	1.79 (1.38–2.32)***
Model R^2^		0.104		
Depressive symptoms			1.04 (1.01–1.08)*	1.04 (1.01–1.08)*
Posttraumatic stress			1.00 (0.99–1.01)	1.00 (0.99–1.01)
Conduct problems			1.05 (1.01–1.09)*	1.04 (1.00–1.09)*
Sensation seeking			1.02 (1.00–1.04)*	1.02 (1.00–1.04)
Perception of risk			0.99 (0.97–1.00)	0.99 (0.97–1.00)
Perception of wrongdoing			0.96 (0.94–0.99)**	0.97 (0.95–0.99)**
Delinquent peers			1.04 (1.01–1.07)**	1.04 (1.01–1.07)*
Parental control			1.00 (0.98–1.03)	1.01 (0.98–1.03)
School attachment			0.99 (0.95–1.03)	0.99 (0.95–1.02)
Teacher support			1.01 (0.98–1.04)	1.01 (0.98–1.04)
Model R^2^			0.192	
Substance availability				1.04 (1.00–1.08)
Model R^2^				0.195

Step 1 included fixed markers of risk; Step 2 added alcohol use in year one to the variables included in the Step 1 Model; Step 3 added individual and interpersonal factors to the variables included in the Step 2 Model; Step 4 added contextual factors to the variables included in the Step 3 Model

*CI* confidence interval, *OR* odds ratio, *R*^2^ Nagelkerke R square

*p < 0.05, **p < 0.01, ***p < 0.001

## Discussion

In this longitudinal study we used a systematic approach to investigate whether a broad range of risk and protective factors assessed during the sixth grade predicted alcohol-related problems when re-assessed in the seventh grade. In particular, individual factors, such as depressive symptoms, conduct problems and a decreased perception of wrongdoing, as well as ethnicity and affiliation with delinquent peers, all predicted more alcohol-related problems in grade seven. The associations remained even when adjusting for having used alcohol by the sixth-grade, which was also the strongest predictor of alcohol-related problems. In contrast, a majority of the contextual and the fixed factors did not predict problems related to alcohol use in the full model. Neither were there any sex- or age-specific associations between the risk/protective factors and problems related to alcohol.

Even though research on risk factors for the use and misuse of alcohol during adolescence is abundant, as yet, comparatively little is known about younger adolescents and alcohol-related problems, especially when examined in a systematic context using a multitude of variables. Our findings are in line with previous studies that reported an association between individual risk factors and problematic drinking. In particular, externalizing symptoms such as conduct problems and affiliation with delinquent peers have been consistently linked to problematic alcohol use [1, 10, 12, 13]. In addition, and contrary to our hypothesis, depressive symptoms were a predictor of alcohol-related problems in the full model. Depression has been theorized to underpin drinking as alcohol may serve as a regulator of negative affect, i.e. to escape or avoid negative emotions [27]. The results of previous prospective studies on depression as a predictor of alcohol use/problems in early adolescence are however mixed [17], with reports of both an association [14] and no association [15], with one study even suggesting a protective effect against early alcohol use [13] which is why it has been argued that internalizing symptoms may be regarded as a protective factor at early ages before drinking becomes normative [16]. However, our finding rather supports previous reports of depression as a unique predictor for substance use and misuse, even at this young age.

Both White and Hispanic ethnicity were associated with more alcohol-related problems. This result partly corroborates findings from the Monitoring the Future (MTF) study conducted in the U.S., even though the students in the MTF study were slightly older, where eighth grade Hispanic students reported more alcohol consumption, being drunk and binge drinking compared to both White and African American students. However, with increasing age both White and Hispanic students reported more alcohol consumption than African American students [20, 28]. In our study, the association between ethnicity and alcohol-related problems remained even when adjusting for other fixed, individual, interpersonal, and contextual risk/protective factors. The commonly reported later initiation and lower rates of alcohol use among African Americans have been linked to African American cultural norms against heavy alcohol use and the higher likelihood of encountering legal problems when being African American [29].

There was no sex-specific difference in the association between the risk/protective factors and alcohol-related problems, nor did boys have a total higher rate of such problems compared to girls. This finding contrasts with previous research that observed greater substance use among males [10]. However, in early adolescence the rates of alcohol use tend to be more equal when compared to later adolescence [30] and in the MTF study girls even reported more alcohol consumption than boys in the eighth grade [28]. We did however find sex differences for individual items, with boys reporting more money problems and school problems in relation to drinking. Neither student-rated parenting behaviors nor the school environment and contextual factors such as substance availability were protective against alcohol-related problems in the final model, indicating that these factors may not be of great relevance for early prevention efforts with regard to problematic alcohol use. This conflicts with previous reports, where parental involvement and a higher quality of parent-adolescent relationships have been suggested as being protective against the negative consequences of alcohol use in adolescence [12]. Rather, when applying a more systematic approach, our findings suggest that preventive efforts should be targeted towards interventions to assess and treat psychopathology in early adolescence, given the importance of depression and externalizing symptoms in the current study, with having used alcohol at age 12 also being an important risk marker that resulted in almost two times higher odds for problematic alcohol use.

Our study has several limitations. First, we used self-reported data, which may have been subject to reporting bias, although adolescent self-reports for some forms of health risk behavior have been found to be generally valid [31]. However, using other sources of data from e.g. parents and teachers would have improved confidence in our findings. Second, the study was limited to a group of deprived American inner-city youth and it is unclear if the results can be generalized to other youth populations. Third, we lacked information on factors that might have been potentially important for the observed associations such as parental psychopathology, family substance use history and prenatal exposure to drugs [11]. Fourth, a multitude of variables were included in the models which may have affected statistical power. However, we had a reasonably large sample with over 400 adolescents reporting alcohol-related problems.

## Summary

The identification of risk and protective factors associated with alcohol-related problems during early adolescence is of great importance given their potentially detrimental effects. By using a systematic approach, we were able to identify several factors which may be targeted for early intervention and prevention programs. Individual factors, such as alcohol use at year 1, depressive symptoms, conduct problems, a decreased perception of wrongdoing and affiliation with delinquent peers, predicted more alcohol-related problems, as did White and Hispanic ethnicity. In particular, interventions directed towards assessing and treating depression and externalizing symptoms, as well as being observant of any alcohol use at a young age, might be especially beneficial in this context.
